# Technopolitical Construction of a River Basin: Turkey’s Encounters and Adventures with the “TVA Idea”

**DOI:** 10.1007/s00048-023-00357-y

**Published:** 2023-05-03

**Authors:** Aybike Alkan

**Affiliations:** TU Berlin, Berlin, Germany

**Keywords:** Development, River basin, Politics of scale, Technology transfer, Southeast Turkey, Entwicklung, Flusseinzugsgebiet, Skalenpolitik, Technologietransfer, Südosttürkei

## Abstract

In the postwar era, the preferred approach to harnessing rivers was through integrated river basin planning (IRBP), which required a holistic focus on the river basin for multipurpose development. While the river basin is taken for granted as the natural unit of development in the definitions of the IRBP concept, this article problematizes the river basin idea and reveals the politics behind what has been deemed natural (scientific), with a specific focus on Turkey’s experience with IRBP. It explores geopolitical and national motivations and challenges in the context of the scaling of the Euphrates-Tigris basin. By approaching IRBP as a process of scale-making, it draws from discussions of the politics of scale in the literature on political ecology, but also incorporates a historical dimension to these discussions with attention to the political and environmental histories of Southeastern Turkey, which became home to Turkey’s first and most extensive IRBP project, the Southeast Anatolia Project (GAP).

The article stretches the chronological boundaries of GAP to the decades prior to the 1970s, when the project was initiated, by analyzing archival materials, including the proceedings of the Grand National Assembly of Turkey, the archives of a daily newspaper, and the expert reports on GAP. The analysis highlights the politics of scale as a powerful constituent of the politics of technological development, and shows the significance of historical analysis to delineate the politics of river basin planning into different layers, including the level of geopolitics, territorial disputes, and international conflicts.

## Abstract (de)

In der Nachkriegszeit war die bevorzugte Methode zur Nutzbarmachung von Flüssen die sogenannte integrierte Flussgebietsplanung (*integrated river basin planning* – IRBP), die eine ganzheitliche Betrachtung des Flussgebiets für eine Mehrzweckentwicklung erforderte. Während das Flusseinzugsgebiet in den Definitionen des IRBP-Konzepts als selbstverständliche natürliche Einheit der Planung angesehen wird, problematisiert dieser Artikel die Flusseinzugsgebietsidee und enthüllt die Politik hinter dem, was als natürlich (oder wissenschaftlich) gilt, mit besonderem Schwerpunkt auf den Erfahrungen der Türkei mit IRBP. Er untersucht geopolitische und nationale Motivationen und Herausforderungen im Zusammenhang mit *politics of scale* im Euphrat-Tigris-Becken. Indem es sich dem IRBP als einem Prozess der Skalenbildung nähert, stützt es sich auf Diskussionen über Skalenpolitik in der Literatur zur politischen Ökologie, und ergänzt diese Diskussionen um eine historische Tiefendimension hinzu. Dabei liegt der Schwerpunkt auf der politischen und ökologischen Geschichte der Südosttürkei, die zum Schauplatz des ersten und umfangreichsten IRBP-Projekts der Türkei wurde, dem Südostanatolien-Projekt (GAP).

Der Artikel dehnt die chronologischen Grenzen des GAP auf die Jahrzehnte vor den 1970er Jahren aus, als das Projekt initiiert wurde. Die historischen Daten des Artikels basieren größtenteils auf den Sitzungsakten der Großen Nationalversammlung der Türkei, den Archiven einer Tageszeitung und den Expertenberichten zum GAP. Die Analyse hebt die Skalenpolitik als mächtigen Bestandteil der Politik der technologischen Entwicklung hervor und zeigt, wie wichtig die historische Analyse ist, um die Politik der Flusseinzugsgebiete in verschiedene Ebenen aufzuschlüsseln, darunter Geopolitik, territoriale Streitigkeiten und internationale Konflikte.

## A Traveling Concept and Its Journey in Turkey, or Integrated River Basin Planning and GAP

In 1977, the Turkish government announced the country’s largest and most extensive regional development project—the Southeast Anatolia Project (GAP). This project was predicated on the concept of integrated river basin planning, which refers to the cohesive and orderly development of water and land within the boundaries of an entire area supplied by a river and its tributaries. Using this concept as a starting point, the planners involved in GAP aimed to harness the Euphrates and Tigris rivers for irrigation, flood control, and the production of electricity. While there had previously been two separate projects, one for each river, they were now considered part of a single basin unit, and GAP was born of the combination of these projects, totaling 13 sub-projects: six for the Tigris, and seven for the Euphrates. These schemes corresponded to the construction of 22 dams, 19 hydropower plants, and an irrigation network for 1.7 million hectares of land. Yet, GAP was a complicated child, whose lineage was described differently by each of the many actors involved, according to their shifting interests. In other words, although Turkish authorities were enthusiastic about the single basin approach, GAP inherited the legacies of previous basin-based debates in terms of worsening international relations and national ethnic conflicts. This article traces the origins of the project with a focus on the idea of the river basin. It asks which kind of political factors deemed the ‘river basin’ as the natural unit of development and how the practices of claiming a specific river basin—the ‘Euphrates-Tigris basin’—were entwined with how high-ranking state actors perceived the political risks and opportunities.

With the dissemination of the concept of integrated river basin planning (IRBP), the river basin became the preferred unit of water development, especially in the post-World War II era. It was based on three major principles: the integration of multiple purposes, basin-wide programs, and regional development, all of which were affected by the definition of the region to be developed (White 1957). Seen in this light, IRBP provided a vision for water development, heavily supported by the iconic example of the Tennessee Valley Authority (TVA), which both materialized and enriched this vision. Although the TVA is usually used as a synonym for IRBP, I suggest approaching the TVA as a specific experience, whereas IRBP can be understood as a lens through which different experiences become visible in response to the specific regions in which it is deployed. As such, IRBP projects can be analyzed within their specific processes of transformation, stimulated by conceptual and empirical advances as well as political, social, and environmental considerations. It is thus key to acknowledge that country-specific settings contribute to diverging understandings of IRBP and foreground different criteria in the construction of river basins.

One of the main purposes of this article is to open the concept of river basin up for discussion, follow it as it spreads across Turkey, and ask how experts, politicians, and intellectuals engaged with this concept and brought together various ideas, techniques, political desires, histories, and basin-based materialities. GAP is a special case for several important reasons: Unlike its predecessor, the development project in the Gediz Valley, GAP was the first regional development project to have an administration of its own (the GAP-Regional Development Administration), similar to the TVA model. Second, since it emerged after the amalgamation of the individual Euphrates and Tigris basins into a single basin, it encouraged discussions on the meanings of the basin and IRBP, and invited a deeper understanding of the concept. Finally, its implementation in a politically contentious region of the country provides fertile ground to highlight how the concept of IRBP and the technical language it provided can be utilized to hinder political action around the basins, their construction and transformation.

This article traces the moments of making the river basin in the context of GAP through the tensions that arose in planning and implementing the project. The main tension that lay at the heart of GAP was the presence of a large Kurdish population and the accompanying ethnic conflicts in the region that overlapped with the area of the Lower Euphrates and Tigris basin(s). Given that the foundation of the Turkish Republic was based on the exclusion of the Kurdish nationality, Turkish leaders were concerned about Kurdish separatism from the earliest years of the Republic. This fear was reinforced by Kurdish revolts and uprisings that took place between 1923 and 1938 in Eastern and Southeastern Turkey, which were suppressed through violent means that went beyond merely suppressing the revolts. Yet, the state was not always consistent in the policies it crafted in response to political challenges posed by Kurdish communities (Kasaba 2000), and these inconsistencies would be manifested in the establishment of GAP. Nationalist bureaucrats and politicians believed that their efforts to initiate a regional development project could serve two opposing ends, namely both integration and division. On the one hand, identifying the region as different from the rest of the country was a necessary first step to alleviating those differences and integrating the region into the norms and regulations of the Turkish state. On the other hand, the recognition of the East as a distinct region of the country implied a further act of recognition: that of the difference between Kurdish and Turkish ethnic identities and Kurdish claims to the lands of Southeast Anatolia. As Kurdish political activism gained in momentum in the 1960s, Turkey’s State Planning Organization (*Devlet Planlama Teşkilatı*, DPT) even refrained from using the word *bölge* (a Turkish word for ‘region,’ which was used euphemistically to designate Southeastern Turkey, and was favored in leftist and Kurdish circles as an allusion to the long-banned term ‘Kürdistan’) in its third five-year development plan for the period between 1973–1977 (Tekeli 2008). Caught in the quandary of accidentally supporting Kurdish demands by identifying the Southeast as a distinct *region*, state actors moved toward explaining underdevelopment and its associated problems as the result of ‘natural’ characteristics, the solution to which was the use of technology. In that sense, the interventions in the rivers were never merely about rivers and the infrastructure that would intervene in their flows. They were also about social relations, national bureaucratic structures, and governing environments as well as their resources and populations. This sociotechnical perspective is well-established in studies of the history of technology, which have developed a contextual understanding of technological change (Kranakis 2021).

To James Secord, the central question within the history of science is “how and why does knowledge circulate?” Krige takes up this argument for the history of technology and invites us to see technology as a form of knowledge embedded in material objects and practices. Many historians of technology have subsequently focused on the question of circulation, problematized it by emphasizing the role of the social, political, or material constraints that impede this circulation and by paying attention to national borders as well as national and local resources that are mobilized to make the circulation of technologies/knowledges possible (Krige 2019; Engerman & Unger 2009). The complex nature of these circulations and processes of negotiations and compromise between the interests of foreign and national actors are also visible in the literature on river basin planning (Hoag 2006; Klingensmith 2007; Lagendijk 2018; 2019).

The circulation of technology and knowledge in and through Turkey has recently received renewed attention from different disciplinary fields with different empirical focuses. Vast attention has been paid to the flows of expertise, knowledge, and technologies between the United States and Turkey, especially in the context of Cold War geopolitics and Turkey’s modernization processes. The studies on this topic emphasize the role of US technical expertise in shaping knowledge and technology production by elucidating the agency of Turkish actors in negotiating their demands. The particular focus of these studies falls on a wide range of topics, including architectural culture (Bozdoğan 2008); art and aesthetics (Smith 2022); controversies over the flows of machines and experts (Keskin-Kozat 2011; 2013); development policies in rural areas (Hartmann 2020); electrification processes (Tunç & Tunç 2022); knowledge and expertise production in engineering, medicine, business administration, and the humanities (Erken 2019); and tourism and transportation infrastructure (Adalet 2018). Despite the increasing interest in Cold War development processes in Turkey, practices of river basin development in the context of the Cold War period in Turkey remain underexplored. One of the few exceptions is Christina Luke’s work on the water development projects in the Gediz Valley, the so-called Aegean-TVA (2019). While this study builds strong connections between the TVA and its Aegean counterpart, it is hard to find such a detailed analysis on GAP. Several studies have recognized the significance of TVA for GAP, such as Mukhtarov (2009), who highlights that although TVA was a source of inspiration for GAP, the earliest inspiration for Turkish actors was in fact the Dniepr River developments in the USSR in the late 1920s. Stahl (2014) recognizes TVA as the ancestor of GAP; Bilgen (2019) notes that the instigators of GAP took TVA as their model and draws attention to the similarities between GAP and TVA. Çelik (2019) outlines the similarities and differences between the two IRBP projects in terms of their aims, administration, finances, and realization. With its detailed focus on the TVA in the context of GAP, this article aims to achieve two seemingly contradictory goals—emphasizing and problematizing the role of the TVA in the establishment of river basin projects outside the United States. In this respect, it highlights both the importance of TVA in the establishment of GAP and the agency of national actors who selectively used lessons learned from TVA and sometimes adopted approaches that deviated from those employed within TVA.

Moving from the 1930s, when the TVA became a topic of interest in Turkey, to the 1990s, when the implications of the construction of the Euphrates-Tigris basin were most visible, each section of the article reveals the different political layers that contributed to both the evolution of the river basin idea as an object of development and its application to the Turkish context. The first part examines the early encounters of intellectuals, scholars, and experts in Turkey with IRBP through the example of TVA. It introduces the various mechanisms through which TVA became part of both the transformation of river basins around the world and the making of political elites in the postwar era, especially in Turkey. The second part of the article focuses on the early basin-based debates in Turkey in relation to the Euphrates River. These debates also provide the backdrop for GAP’s emergence by revealing the political complications GAP would face in the late 1970s. The final part of the article is devoted to the analysis of the political/economic meanings and material effects of the scientific claims made about the boundaries of the basin. This discussion shows how the process of translating IRBP into Southeastern Turkey was enmeshed within a network of various interests and the implications of that translation. In conclusion, the article contributes to recent discussions on technology transfer in the literature on the history of technology, that consider transfer, not as a process of adoption or imitation but rather one of adaptation. It does so by introducing an overlooked case from Turkey and examining the role of politics at global, national, and local scales in this process of adaptation.

## The Interest in the TVA in Turkey: River Basins as an Object of Development

Although the consolidation of the idea of integrated river basin planning was a long-term process in which numerous experts and engineers from different parts of the world participated, the dissemination of the idea was mostly realized through the TVA. Especially in the postwar era, TVA demonstrated both how existing technology could be deployed to exploit nature and how to build and use this capacity through its training programs. While dams occupied a central place in US geopolitical strategies for the expansion of capitalist development against the threat of communism, state elites in the Global South believed that hydroelectric and irrigation infrastructure would propel their states through the various stages of modernization. Epitomizing the ambitions of foreign technical assistance, TVA thus became essential for the spread of development in the countries of Africa, Asia, and Latin America. The handbook of regional development schemes published by the TVA Technical Library in 1961 included river basin planning examples from 34 countries across five continents, all of which were developed in connection with TVA (Martin 1970).^1^

As Roscoe Martin (1970) argues, Lilienthal’s book, *TVA: Democracy on the March*, played a major role in the international reputation of the authority. Published in 1944 and translated into 19 languages by 1970, the book was “a veritable barrage of information and inspiration, not to mention a spirited invitation to emulation” (ibid.: 8). Another factor fueling the demand for assistance from TVA was the promotion of the agency by technical aid institutions such as the US Agency for International Development (USAID), the United Nations (UN), and the US Department of Agriculture (Lagendijk 2019). Most of the visitors to TVA came under official or semi-official sponsorship, and 25 percent of these trips were arranged by USAID, the largest consumer of TVA’s technical assistance and the main agency directing TVA into this area (Martin 1970: 20–22). As the rest of this section will show, Turkey—with its huge basin-wide program—was a significant example among the countries receiving assistance from TVA.

In the 1920s and 1930s, Turkish state leaders built close relationships with their Soviet counterparts; in response, Moscow not only advised Turkey’s five-year development plan but also sent equipment and engineers to accelerate Turkey’s industrialization process (Hirst & İşçi 2020). In one example of interwar Soviet-Turkey cooperation, the two countries signed agreements on the Aras River, which delineated one section of the border between Turkey and the Soviet Union, including one agreement on the construction of the Serdarabad dam (İşçi 2022). Although the expert networks around river basin planning were mostly established after World War II, when Turkey and the Soviet Union were at odds and the former turned to the United States, the concept started to spread in Turkey as early as the 1930s, mostly through the work of Turkish intellectuals interested in the experience of TVA. In 1938, Yunus Nadi, founder of the oldest national newspaper, *Cumhuriyet*, highlighted the achievements of TVA and how Turkey could learn from the TVA’s achievements. He argued that Turkey had better conditions than the United States for the establishment of a TVA-like project, due to its cheaper labor costs and less hilly ground. In addition, since the Tennessee Valley region of the United States was even less civilized than Turkey, Turkey would benefit even more from the initiation of such development projects. Referring to TVA-led infrastructural development, he added “these are neither empty words nor dreams, these are realities dollarized and concretized in the Tennessee Valley, I hope my beloved friends returning from America with new knowledge and energy will be dutiful to their country and nation” (Nadi 1938, all translations from Turkish by the author). American-educated Nusret Köymen, a specialist in the areas of sociology of education and village development, also glorified TVA as a democratic regional planning institution superior to the repressive total planning organizations of the Soviet Union. According to Köymen, Roosevelt’s TVA was “the most valuable gift from America to world civilizations” (Köymen 1948). In this respect, the interest in TVA in Turkey was one way to demonstrate one’s political, especially anti-communist leanings, mirroring American efforts to promote TVA as a “counter-model to communism” (Gilman 2003: 39).

During the Cold War, Turkey became a significant ally of the United States, who was interested in its strategically important water routes and borders with Soviet Russia in the East and with Soviet Bulgaria in the West. Due to its location and resources, Turkey was perceived as a barrier against Soviet expansion into the Middle East (Rustow 1987: vii). In 1952, George McGhee, the US Ambassador to Turkey, said to President Celal Bayar that “Turkey was the natural leader of the Middle East because of her historical position, military strength, political stability, economic development, and membership in NATO” (Adalet 2018: 45). Turkey’s ostensible leading position in the Middle East made the development of the rivers an important issue for the country: UN conference reports underline that if the rivers were developed in accordance with technical conditions, the people of Turkey, Syria, Iraq, and Iran would all benefit in terms of hydroelectricity production, the procurement and export of petroleum, and the transformation of wastelands into cultivable lands (*Cumhuriyet* 1949). Moreover, in their article on the international applicability of TVA, Bochenski and Diamond (1950) claimed that harnessing the Euphrates and Tigris for irrigation and electricity would bring wealth to Middle Eastern landscapes that had long suffered from drought and low rainfall. They happily noted that Turkey began to appreciate the implications of total basin development as information about TVA spread throughout the country (ibid.: 68).

In the 1950s, Turkish universities began to host exhibitions and conferences on TVA, including one introducing the irrigation and electricity generation activities of TVA held by Ankara University’s Political Science Department in 1955 (*Cumhuriyet* 1955a). Two years later, the university organized a conference on regional planning as part of its celebration of the Second Annual Settlement and Urbanism Week (June 5–7, 1957). The conference was opened by the Technical Co-Director of the UN-Egyptian Institute of Public Administration in Cairo, Richard Niehoff, with a presentation on his experiences working for the TVA. After underlining the Authority’s official a‑political stance, Niehoff ended his presentation by drawing attention to the Euphrates and Tigris as the two large river systems suitable for TVA-like projects (Geray 1965). In line with the notion of river basins as the natural unit of water management, the importance of the Euphrates and Tigris was usually ascribed to their natural characteristics. Yet, no matter how natural the units of development in question were, the river basins that US technical assistance programs targeted for TVA-like projects were invariably of (geo)political importance to US foreign policy—such as the Euphrates and Tigris basins in Turkey.

As the idea of river basin planning became popular in Turkey, technical assistance programs sought to establish an institutional setting that could undertake such activities. Before the 1950s, the Electrical Power Resources Survey and Development Administration (Elektrik İşleri Etüd İdaresi, EİE) was the responsible body for river management in Turkey. As correspondence between TVA and Turkish citizens increased and TVA officers organized tours of the Norris Dam, the Chattanooga Dam, and the Widows Creek Steam Plant for Turkish engineers during this period (Luke 2019: 96), the EİE had only a few water engineers and a very limited budget, making it impossible for the administration to conduct comprehensive basin research. Such extensive research only became feasible after the creation of the Turkish State Hydraulic Works (Devlet Su İşleri, DSİ) in 1954. The DSİ was modeled directly on the US Bureau of Reclamation (USBR), a domestic agency of the US Department of Interior responsible for developing water infrastructure (Sneddon 2015). DSİ officials writing the official history of the institution acknowledged, with reference to the USBR, that Turkey was half a century behind the United States. Yet, they were still pleased by the DSİ’s growth and competency, which they believed was directly connected to the successful adoption of the institutional structure of the USBR and their continued close relationship (Yıldız 2014).

Turkey’s relationship with the USBR began in the early 1950s under the sponsorship of the Mutual Security Agency (Sneddon 2015: 182). In those years, Turkish engineers were sent to USBR offices in Denver for technical training, and a team of USBR engineers was sent to Turkey to help translate the techniques of river basin planning and advise on the institutional structure of the DSİ (ibid.). Orhan Güncüoğlu, engineer and deputy general director of the DSİ from 1955 to 1960, claimed that a group of DSİ engineers visited the USBR annually (Turgut 1992: 145). In addition to training experts, the United States sent a considerable volume of machinery and technical equipment as the result of an agreement made with the Turkish Minister of Public Works in 1955 (*Cumhuriyet* 1955b). This direct engagement between the technical institutions of the United States and Turkey lasted until the late 1960s, both strengthening Turkey’s interest in the TVA model and enabling the dissemination of policy prescriptions designed in the United States for adaptation into the Turkish context.

**Fig. 1:**
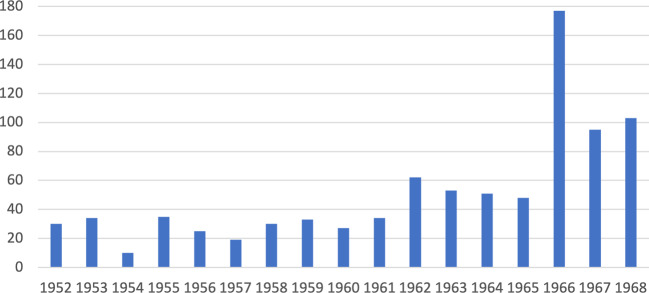
Number of visitors to TVA from Turkey by year (data supplied by Martin 1970)

During this time, Turkish engineers and experts also received direct technical assistance from TVA personnel. Among the practices of direct technical assistance, foreign visitor programs were the most common, wherein professionals and technicians from other countries came to TVA offices for two weeks or more for special training. While these visitors were TVA’s first involvement in international assistance before World War II, and a considerable number of experts continued to visit TVA during the War, the number of visitors increased dramatically in the postwar period, quadrupling between 1945 and 1948 to 686 visitors, and reaching over 1,000 visitors by 1950 (Martin 1970: 28). Between 1952 and 1968, eight countries (Brazil, Taiwan, India, Japan, Mexico, Sweden, and West Germany) sent more than 1,000 visitors to TVA, mainly to Knoxville, while visitors from Turkey, Canada, Colombia, England, Indonesia, Italy, Korea, Thailand, and Yugoslavia constituted the second major visitor group with between 500 and 1,000 visitors per year (see Fig. 1 for the statistics for Turkey).

**Fig. 2:**
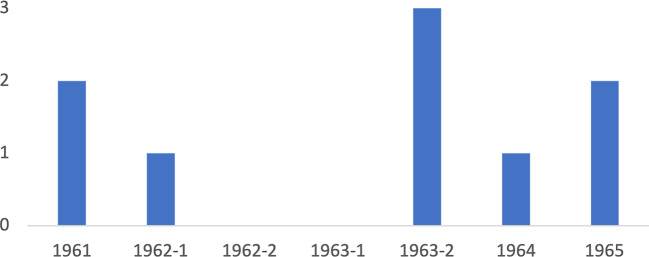
Participants from Turkey in the Resource Development Seminars (data supplied by Martin 1970)

Although the directors of TVA were satisfied with the heavy foreign traffic, it was not free of concern. TVA Director of Information Paul Evans, concerned about visitors’ inability to “get practical, overall knowledge of methods, problems, and pitfalls,” announced a five-week experimental resources development seminar (cited in Martin 1970: 134). This led to seven content-rich seminars in Knoxville, Tennessee, between 1961 and 1965, some of which were supported financially by USAID. In these five-week seminars, participants were instructed by both TVA executives and specialists; they watched films on the Tennessee Valley and TVA; conducted valley-wide tours to selected dams, steam plants, and local industrial sites; and were informed about the TVA’s organization, procedures, and activities (Martin 1970: 136). Of the 108 visitors in total, nine were from Turkey, the third-highest number of visitors (ibid.:138) (Fig. 2).

The more extensive TVA programs, such as the five-week resource development seminars, were aimed at government officials and employees who had or had the potential to rise to high-ranking positions in their governments. This aim was congruent with American geopolitical strategies to expand capitalist development against the threat of communism, and as such, TVA had a major role in the creation of the political elites and technocrats of the postwar era: The professionals visiting TVA not only contributed to the wider acceptance of the Authority throughout the world but also occupied important positions in their home country after their return.

Süleyman Demirel, who had built strong relationships with the USBR and TVA before serving as prime minister and the ninth President of Turkey (1993–2000) is a case in point. After graduating from the Civil Engineering Department of Istanbul Technical University, Demirel began working at the EİE in May 1949 as a project engineer. Soon thereafter, the institution sent Demirel to the United States for nine months. This acquaintance with the US experience in the early years of his career allowed Demirel to imagine the path that he believed Turkey should follow. Regarding his first visit to the United States, he said, “the United States had reached the apex of water engineering in the western states. My primary ideal was to find an opportunity to apply the things I had seen and learned there” (cited in Turgut 1992: 109). That opportunity would come after his second visit to the United States on an Eisenhower Fellowship, a program established to further the goals of the Eisenhower Doctrine, which came out of US fears of Soviet expansion in the Middle East. During his fellowship, he visited TVA and engaged with the work of TVA personnel. Upon his return in 1954, Demirel became the head of the Dams Administration at the DSİ. A year later, he became General Director, a position he held for six years until the 1960 coup.

As the General Director of the DSİ, Demirel “was instrumental in securing a resident team from Reclamation [USBR] that assisted him in moving forward with an ambitious water program” (Ives & Bochar 2002: 672). In 1959, he sent an official letter to the Economic Cooperation Administration in the United States about the administration of technical assistance, in which he asked to send DSİ personnel to the United States to increase their knowledge in different areas of specialization. Demirel also attempted to solve the language barrier for Turkish engineers by requiring potential visitors to attend an English course for six months before going to the United States (Turgut 2005: 44–5). Demirel’s activities at the DSİ, which were mostly concentrated on the development of the Euphrates basin, were interrupted by the 1960 coup, after which Demirel began his military service in the State Planning Organization. After the completion of his military service, he worked as a consultant engineer for the American engineering and construction firm Morrison Knudsen, a major US corporation involved in the construction of the Hoover Dam that epitomized the technical prowess of US engineering at the time. Due to his work there, his opponents nicknamed him “Süleyman Morrison Demirel” or the “American stooge” (Vali 2019). Turkish leftists in particular used these nicknames to denigrate Demirel, to claim that he was acting in the service of American imperialism. Yet, this opposition was not an obstacle for Demirel, who, in 1964, became the chairman of the newly established Justice Party (Adalet Partisi, AP), a center-right party founded as a successor to the Democrat Party, and then the youngest prime minister of Turkey in 1965. His career path was similar to those of other TVA visitors who would go on to hold important political positions upon return to their home countries, revealing the effect of TVA on creating the technocrats of the postwar era.

As Wiebe Bijker argues, the meaning of the label ‘technocrat’ changed as elite visions of national development shifted during the postwar period. This period witnessed the emergence of a new elite group that applied technical expertise to the processes of political decision-making. The agents of development were thus these new elites who imaged social change through technical industries and laboratories rather than through parliamentary action (Bijker 2006). Consistent with Bijker’s claims, Turkey produced “a whole stratum of technocrats, many in the American mold, exercising power through an ever-expanding postwar bureaucracy” (Stahl 2019: 40). In addition to Demirel, the most prominent members of this group were those in Demirel’s circle in during his time at university: Süleyman Demirel, Necmettin Erbakan, and Turgut Özal were all engineering students at Istanbul Technical University in the late 1940s, taking part in international expert networks, who would later become center-right prime ministers and presidents of Turkey. Trained as engineers, they each reflected Bijker’s definition of technocrats and played crucial roles in the transformation of the Euphrates and Tigris rivers. Ultimately, river basin planning in Turkey was a process that intertwined the geopolitics of the Cold War and Turkey’s national political agenda, which complicated the relationship between TVA and its Turkish counterpart.

## The Making of the Keban Dam: The River Basin as the Locus of the Euphrates’ Development

### Early Basin-Based Discussions About the Euphrates

In his report to the TVA Board of Directors, entitled “TVA and International Technical Assistance,” Roscoe Martin, one of the pioneers of the academic discipline of Public Administration in the United States, asks:

“Is the Tennessee Valley Authority exportable? The answer is a clear and unequivocal ‘No.’ Few responsible American students of TVA have ever expressed the opinion that it is. Is the TVA idea transferable? This question differs in important respects from the one asked before. The answer here is a guarded ‘Yes,’ provided it is understood clearly what is (and what is not) being transferred and provided further that favorable conditions prevail in the country proposing to import TVA” (1970: 132).

Here, Martin emphasizes the distinction between TVA and the TVA concept. In his words, “the TVA idea rests upon one simple (in truth all-enveloping) concept: the multiple-purpose development of the natural resources of a region” (ibid.: 132). In any given setting, this idea would be made manifest in new ways, more and less similar to the target set at the beginning. Related to this point, Martin draws another distinction in terms of the mechanism through which this process operates—between transfer and the impossibility of exporting the TVA idea. Here, Martin introduces the notion of transfer to highlight how the TVA idea is not an exportable good, but one to be interpreted by various individuals, immersed in different material conditions. These distinctions are significant vantage points from which to explore the relationship between TVA and other IRBP applications guided by TVA-adjacent actors.

Among the things transferred as part of the “TVA idea” was the transfer of a “powerful geographical ideal:” the river basin as the most appropriate unit for water development and management activities (Sneddon 2015: 4). In fact, river basins were considered an effective unit of administration as early as the eighteenth century. French cartographer Philippe Bauche, for example, defined the river basin as “the set of all the slopes on which fall the waters that converge to a same river or creek” in the mid-eighteenth century, and pioneered the idea that the world is divided into lands and regions (Hartshorne 1939 cited in Molle 2009: 485). In the nineteenth century, topographers used river basins as the unit of analysis to determine the total annual discharge of a river, although these measurements were not translated into regional development efforts and water development remained based on local watersheds (Wescoat 2000: 151). As another example, British Civil Engineer Sir William Willcocks, who was born in India and transferred his experiences in India to North Africa,^2^ dreamed of harnessing the flow of the entire drainage basin for multiple purposes and made plans for the Euphrates, Tigris, and Nile basins by understanding them as unified systems (White 1957: 163). These early deliberations would become concrete institutional schemes in the early twentieth century. In 1921, the French state created the National Company of the Rhône which was charged with developing and managing the river along its three-hundred-mile course from the Swiss border to the Mediterranean Sea. Spain also established river basin authorities that were the public bodies responsible for waterworks and management under the dictatorship of Primo de Rivera from 1923 to 1930 (Embid 2003). In this regard, while Martin’s differentiation between TVA and the TVA idea is valuable in terms of directing our attention to what is being transferred, one should acknowledge that the history of the TVA idea is actually the history of the development of IRBP as a concept and practice for water development. TVA was thus the result of the theoretical thinking of at least three preceding decades in the area of water development and management (Teclaff 1967); it was also the source of IRBP’s wider dissemination far from Europe in the 1950s and 1960s.

As the concept of the river basin was becoming increasingly popular in the postwar era, Turkey’s newly established State Hydraulic Works (DSİ) began to organize its water development activities based on the idea of the basin. In 1954, the year of the DSİ’s establishment, Turkey was divided into 26 basins, including the Euphrates and Tigris basins. Immediately following its creation, the DSİ began investigations into the Euphrates and Tigris (Turgut 2000). These two rivers were appropriate for IRBP for several reasons. First, data collection on their flow patterns dated back to the 1930s, providing relatively long-term information on fluctuations. Second, the rivers were not already dammed, and were thus suitable for creating a design from scratch, in line with the principles of holistic planning. Third, according to the UN Economic and Social Council’s research, Turkey had a hydroelectricity potential of 500 billion kilowatt hours, 70 percent of which could be produced by the Euphrates and the Tigris (TBMM 1962). The rivers thus had the potential to bring development to the most economically underprivileged region of Turkey—Eastern and Southeastern Anatolia.

Although both rivers were recognized as significant resources for development, the main focus fell on the Euphrates. In 1961, the Euphrates Planning Group Authority (*Fırat Planlama Grup Amirliği*, FPGA) was established to coordinate the studies conducted on the Euphrates valley. The Authority’s first project was the Keban Dam project. In the 1930s, before the establishment of the DSİ, the EİE paid several official visits to the Euphrates. Two water engineers, Necip Suveren and Abdullah Orton, and a cadaster technician, Celal Şar, headed expeditions along the Euphrates and marked the narrow passage at Keban as a suitable place for dam building. In response:

“The administration initiated geological and topographical surveys in the Keban pass in 1938, and after two years, the mapping studies for the Keban catchment area [the area of land bounded by watersheds] were completed. … As the studies on the Euphrates continued, the EİE sent one of its young engineers to the United States to allow him to gain knowledge and good manners” (Turgut 1992: 48).

This young engineer was Süleyman Demirel, who started to work as a project manager on the construction of the Seyhan Dam after his return from the United States. After his second visit to the United States, he was inspired to build a dam such as Keban:

“In 1949, I was sent to the United States for a year. I visited Western states, Eastern states and observed the studies related to my profession. What I saw there both encouraged and sickened me. Every work of civilization made me sad, and I asked, ‘why does my country not own such monuments?’ I cannot forget … I observed that monument [the Hoover Dam] for three days. Every morning, I visited the dam and watched it, while sitting on a rock. We, ourselves, have built that kind of a large monument by starting the construction of the Keban Dam. Whenever I fly over the Keban Dam or make my way there, I connect these two dams to each other” (Süleyman Demirel, cited in Turgut 2005: 24).

The US involvement in the making of the Keban Dam was not limited to being a source of inspiration. During the planning process, the EİE built professional relationships with the American consulting firm EBASCO Services Inc.^3^ Within the context of USAID’s aid programs (which were the main sponsor of the TVA and USBR’s international activities), in 1959, Turkey invited EBASCO to conduct research on the energy potential of the country that could feed into Northwest Anatolia interconnected electricity system (Eriç 1962). EBASCO’s main goal during this process was to prove the necessity of the project and consult on how the project should be carried out using EİE data and reports. After approximately two years of work based on EİE reports and data, EBASCO submitted its own report in March 1961. The report claimed that the construction of the Keban Dam was indispensable to Turkey’s increasing electricity demands, and, moreover, that the Dam should be operational by 1968. Following the feasibility studies, EBASCO continued its involvement in the project, providing various services such as conceptual design, procurement, financial services, and construction management (APA 1982).

While the EBASCO report was in line with the EİE’s expectations, the planning of Keban Dam raised concerns about the future design of the Euphrates basin. Certain national engineers involved in the Keban project, mainly US-educated experts working at the DSİ, were well aware that the project required a holistic approach to the river basin. The entire basin had to be planned simultaneously, because if the first dam in a basin was inappropriately built, it would have a downstream effect on the whole basin’s ability to produce hydropower and irrigation. In early 1962, Daniyal Eriç, Energy Commission Director at the DSİ, published an article in *Cumhuriyet *stating that:

“The studies that have been completed thus far on the Euphrates river basin merely concentrated on the Keban pass… Modern methods treat the whole river basin from the source to the river mouth as the smallest unit of research… It is useless to prepare detailed projects for a single dam without producing a holistic study and reaching precise conclusions, because those projects may undergo significant changes, the location of dams may change and even the projects may be withdrawn after the process of river basin planning has begun” (Eriç 1962).

While Eriç advised postponing the Keban Project until the planning of the entire basin was completed, other experts believed that the project could be implemented in parallel to the wider basin research. The commission report on the “Keban Dam and the Lower Euphrates Basin Development Project” prepared by a committee of deputies on behalf of the parliament also recognized the dam as the first step of an IRBP project, but still underlined that the planning of Keban could be completed simultaneously with the collection of the required data to build other dams on the Euphrates. The report clearly stated that the electricity potential of the Euphrates could be harnessed by building three or four dams, which could only be planned for after regulating the flow of the river by building the Keban Dam (TBMM 1962: 361). As the name of the report suggests, the experts considered the lower section of the Euphrates River as a separate unit, albeit dependent on interventions made in the upper section. Recai Kutan, who was the DSİ Regional Director responsible for the Euphrates and the Tigris basins in the late 1950s and 1960s, noted that DSİ personnel had not understood Keban as part of a single project because they could only irrigate the Lower Euphrates region by regulating the flow in the Keban Dam reservoir (Kutan 2001). This meant that the dam was the “necessary first project,” as articulated in 1964 by engineer Korkut Özal, Demirel’s colleague from Istanbul Technical University (Stahl 2019: 46). Despite their different views on the timing of the project, the experts were in agreement both on the importance of Keban for the development of the “Lower Euphrates Basin” and on the need to expedite the research around the basin by strengthening institutional infrastructure.

The difference between TVA and the TVA idea, or its export and transfer, became obvious during the transformation of the institutional processes involved in river basin development in Turkey, as Turkey would apply the TVA idea with a quite different institutional base. To create a TVA-like institutional setting and speed up the preparation of the Lower Euphrates Project, the commission report on the “Keban Dam and the Lower Euphrates Basin Development Project” proposed the formation of a new single authority. It stated that,

“the US Tennessee Valley Authority is the best example showing the achievement of such authorities in developing a backward region. We believe that it will be a very useful maneuver to carry out the development studies of the Euphrates basin with an organization such as ‘the Euphrates Basin Authority’” (TBMM 1962: 363).

**Fig. 3:**
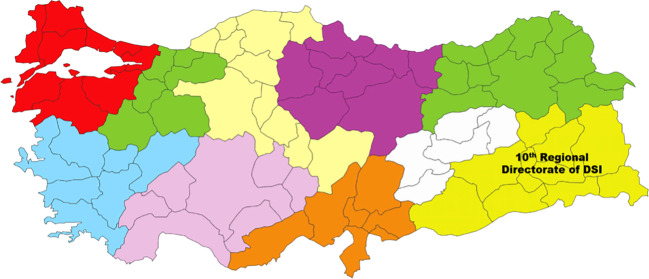
Regional Directorates of the DSİ in 1954

When this report was released, the Euphrates Planning Group Authority (*Fırat Planlama Grup Amirliği*, FPGA) already existed—established in 1961 to realize the Keban Dam project and coordinate the studies conducted on the Euphrates. Yet, its organizational structure was problematic from the beginning. Firstly, the FPGA was located in Diyarbakır province, despite the fact that the flow of the Euphrates does not pass through Diyarbakır. Since the development of the Euphrates and the Tigris was the responsibility of the 10th Regional Directorate of the DSİ, whose directorate was based in Diyarbakır, Diyarbakır became the official center of river basin studies for a region encompassing the provinces of Mardin, Siirt, Bitlis, Muş, Van, Hakkari, and Urfa (See Fig. 3). Thus, the formation of the Authority involved nothing more than adding an additional structure; it did not change the overall organization of waterworks around the basin. Secondly, and more importantly, unlike the TVA which replaced the authority of the USBR and assumed full responsibility for the development of Tennessee Valley, the FPGA was established under the DSİ. Although such an organization was not expected to solve the problem of coordination between different organizational bodies in Turkey, including the DSİ, the EİE, and the ministries of industry and finance (Ardıçoğlu 1963), numerous bureaucrats and politicians had strong reservations about creating regional (semi)autonomous structures as they equated regionalism with separatism, or at best federalism (Geray 1997: 299). The fact that the Euphrates and Tigris flowed through the regions rife with Kurdish dissent and resistance exacerbated the separation anxiety in the context of GAP.

### Political Challenges in the Creation of the Euphrates Basin

The roots of the territorial anxiety and the accompanying separation fears can be traced back to the Treaty of Sèvres, which granted the Kurds an independent state in Southeast Anatolia. Although the treaty was never implemented, due to its rejection of the Turkish national movement which fought and won the War of Independence, Kurdish claims on East and Southeast Anatolia endured and imposed “political and psychological boundaries” to be tackled by the state leaders (Gorgas 2009). In the first years of the Republic, those claims were manifested through the revolts of Sheik Said (1925), Ağrı-Dağ (1927–1931), Dersim (1936–1938), and others between 1923 and 1938. Among the various responses by state leaders to these revolts was the *Şark Islahat Planı* (Eastern Reform Plan, 1925), which went beyond merely responding to these revolts: The plan clearly stated that the East bank of the Euphrates River was entirely Kurdish and should be Turkified after reinforcing Turkish identity on the West bank of the river (Bozarslan 2009: 342). The plan took its inspiration from the 1924 Constitution, which prohibited the cultural rights of non-Turkish ethnic identities, and became the guiding document for Republican policies against the Kurds (Yeğen 2011: 168).

While the following decades did not witness such widespread revolts, the ethnic conflict became more intense with the rise of the PKK (Partiya Karkeren Kurdistan, the Kurdish Workers’ Party), whose members led an armed struggle for an ethnic homeland in Southeastern Turkey. The PKK conducted its first armed attack in 1984, and escalated its attacks in the 1990s, parallel to mounting state violence in the Eastern parts of the country. Given the conflict between Turkish and Kurdish armed forces, making any claim on Southeast Anatolia, even in the context of development, became more dangerous than ever. This further reinforced the waning use of the word ‘region’ as it evoked the idea that Turkey could be separated into regions and thus lose those regions to various ethnic communities. In short, the first and most extensive regional project in Turkey was planned and implemented in a political environment where the connotations of ‘region’ were a significant obstacle to any regionalism, even in the context of regional development.

Indeed, looking at how the Keban Project was discussed in the 1950s and 1960s reveals Turkey’s ethnic conflicts and the question for state elites of how to treat the Kurdish population. All ruling parties in Turkey faced the difficulty of governing the East and reflected their fear of separatism in parliamentary discussions. When the supporters of the project framed Keban as part of the development of the East, they were thus accused of pursuing regionalism, which implied separatism. For example, Şevki Erker, deputy of the center-right Democrat Party (Demokrat Parti, DP), said “Eastern provinces are merely a geographical expression. There is no difference between the East and West of our homeland… All provinces struggle for the same heart and the same soul (TBMM 1958).” This attitude persisted into the 1960s, as the development of the East through river basin planning remained on the agenda. When the construction of the Keban Dam became an issue in parliamentary budget discussions, the regionalism question pervaded public discourse. After the acceptance of the budget proposal for Keban’s construction, certain politicians resigned from the budget commission, claiming that the decision went against the nation’s stabilization policies and the principles of state administration (*Cumhuriyet* 1962). *Cumhuriyet* reported the budget decision under the heading of “Is the regionalism mentality rising from the grave?” (ibid.).

The accusation of regionalism and the effort to explain differences in terms of geography were one of the rare topics on which politicians from opposing parties agreed. For example, a minister from left-leaning Republican People’s Party (Cumhuriyetçi Halk Partisi, CHP), which came to power after the DP government was ousted during the 1960 coup, made similar arguments as his former DP colleagues, stating,

“it is not right to distinguish citizens as Eastern or Western, we should not disregard national unity. Nobody should take offense. What I say is that we should not provoke specific incidents by making the argument that some parts of the vatan [homeland] receive fewer services from the state, marking it for political exploitation” (TBMM 1964).

The underdevelopment of the East was thus dangerous, not only because it broke up the unity of the nation into East and West, but also because it made the state accountable for this underdevelopment. Therefore, even when the deputies acknowledged the regional imbalance, they were cautious in framing its source, putting forward the notion that underdevelopment was a geographical problem (in the same manner as Erker’s claim that the East is only a geographical expression). Deputy of the right-wing Justice Party, a descendant of DP, led by Demirel, İlhami Ertem spoke to this point, when he argued that: “there is no difference between the Eastern provinces in terms of investments for the services of transportation, water provision, etc. But there is a reality, there is a difference between the East and West and it is all because of *natural conditions*.” (TBMM 1964, *emphasis added*). When the development of the East or more particularly, the implementation of the Keban Project was at stake, this discourse acted as a means of legitimation by erasing the social and political connotations of the East and reframing the construction of the dam as the remedy for the economic underdevelopment of Eastern Turkey resulting from its poor natural conditions. The displacement of any political and social realities with natural/geographical ones within state discourses also allowed high-ranking bureaucrats to legitimize their large-scale infrastructure project on the basis of compensating for natural disadvantages through the deployment of technical means.

When Demirel was elected as the prime minister in 1965, one of his government’s first actions was to declare the implementation of a ‘special’ plan for Eastern and Southeastern Turkey. In order to soothe the opposition’s accusation that this special plan was based on separatism, Demirel stated:

“We declared that we will prepare a special plan for the development of the East and Southeast regions of Turkey. Our aim is not to divide Turkey but to accelerate its development. Is it separatism if this special plan brings new roads to Eastern and Southeastern provinces that are deprived of them? If we carry electricity for the welfare of the population, is this separatism?” (cited in Turgut 1992: 124).

Demirel’s viewpoint was also congruent with those of the experts advocating the construction of the dam before the planning of the entire Euphrates basin was completed. According to him, the Keban Dam was the key facility on the Euphrates, and after its construction, it would be easier to assess the potential downstream. Accordingly, the construction of the Keban Dam began in 1966, one year after Demirel’s election. As with the planning of the project, its implementation was made possible through the use of politically facilitated expert networks. A French-Italian consortium, SCI-Impregilo, took responsibility for the construction after the funding for the project was secured from the German, Italian, and French governments. During construction, Demirel and other experts around him continued to emphasize the importance of Keban in relation to the integrated development of the Euphrates. At the signing ceremony for the Keban contract on February 19, 1966, Demirel demonstrated his enthusiasm:

“Today, I hope that imagining a Ruhr system, a Tennessee system, or Niagara system around the Euphrates with mines under operation and a developing industry is a dream that nobody finds exaggerated; because today we witness the realization of the things that we could not even have imagined ten years ago. I am saying this because you should not understand Keban just as Keban. We have a piece of land in our country called Southeast Anatolia. People are waterless there… If those lands are irrigated, it is possible to harvest crops twice a year. Southeast Anatolia is one of the most productive areas in Turkey” (Turgut 2000: 95).

A few months later, this time at the commencement ceremony for the dam, Demirel again mentioned that Keban was just the beginning of the development of the Euphrates (Turgut 2000):

“Today, we can only access one-fifth of the Euphrates’ electricity potential. Before this construction is completed, we need to start another project that will give as much electricity as Keban. And before that one is completed, we need to start the construction of the third one. And in the meantime, we should think about the irrigation of the lands between the Euphrates and the Tigris, about the facilities that will irrigate those lands” (Turgut 2000: 99).

Indeed, the planning and construction of the Keban Dam was conducted in parallel with the studies for subsequent projects on the Euphrates. Three years after its creation, in 1964, the FPGA prepared the “Reconnaissance Report for the Euphrates Basin” (*Fırat Havzası İstikşaf Raporu*) assessing the energy and irrigation potential of the basin. The report proposed the construction of 80 dams, 66 hydroelectricity plants, and networks of irrigation, which would allow the irrigation of 1.6 million hectares of land and an annual production of 23.87 million kilowatt-hours of energy (Turgut 2000: 86–7). The “Reconnaissance Report for the Lower Euphrates Basin” (*Aşağı Fırat Havzası İstikşaf Raporu*) followed in 1966. Given that the planning of the Keban Dam was completed, and the project was ready for initiation, the report specified the subsequent dam projects that would be realized in the region between Keban and Turkey’s border with Syria. During and after the preparation of these reports, the number of TVA visitors from Turkey increased dramatically, and Turkey became the country with the largest number of visitors to the TVA in 1966, 1967, and 1968.

As the rapid preparation of these reports implies, the construction of the Keban Dam was just the beginning of the activities around the Euphrates. The dam provoked debates on the boundaries of the river basin, and led to an understanding that the lower part of the Euphrates could be considered holistically (as the expression ‘Lower Euphrates basin’ demonstrates). The following projects on the lower part of the Euphrates would be based on a single plan—as the idea of integrated development required. Maybe the plan was not the ‘best plan,’ but surely it would be the largest and most ambitious project in Turkey. Almost a decade after the inauguration of Keban, the DSİ came up with its next ambitious project, GAP, and Keban began an important signpost in the history of GAP by making the project possible in terms of its technical requirements. As Nusret Özgen, one of the early technicians working on the Keban Dam, acknowledged, Keban was a school, a space of knowledge and experience for a young cohort of engineers from Turkey; they learned a lot from foreign engineers during the construction of the dam (Taşkın 2015: 40). It was also a space through which the technopolitical networks of water development were constituted, and Turkish technocrats gained experience in dealing with relevant foreign and national actors. In addition to these technical/managerial contributions, the Keban Dam deserves attention in the analysis of GAP as it sheds light on the path leading to GAP, as well as hinting at the political fault lines the project would be constructed on.

## Between the Two Rivers: River Basins as a Space for Technical Claims and Contestations

The Euphrates and Tigris are two different rivers that have been mentioned together since ancient times. They share certain important characteristics, such as having extremely variable flow regimes, which have affected cultivation practices due to the danger of floods during the harvest. For this reason, ancient rulers and large landowners attempted to construct dikes to prevent floods from destroying their crops. While they were able to control the Euphrates to a significant extent by building escape channels into the desert, they were not able to regulate the Tigris due to the rapid flows coming from its tributaries (Kangarani 2005). As large territorial empires became preeminent in the first millennium BC, the Tigris and Euphrates underwent numerous changes in flow due to the installation of gigantic irrigation canals (Husain 2021). Efforts to harness the rivers’ fluctuating flows took a different turn in the Ottoman era, when Sultan Süleyman I (1520–1566) merged the rivers under a unified drainage basin as part of his imperial project in the East (Husain 2021). At the beginning of the eighteenth century, natural and human disasters forced the empire to restore order around the river basins, which resulted in the emergence of Baghdad as an administrative center in the region (ibid.). Nineteenth-century Ottoman administrators such as Mehmet Reşit (1852–1857), Mehmet Namık Pasha (1861–1868), and Midhat Pasha (1869–1871) all worked to regulate and control the flow of the rivers (Stahl 2014: 4). Even though their work also included the improvement of navigation and irrigation, each goal of river development was handled separately since the idea of multi-purpose development had not yet come to the fore.

This separate focus on the Euphrates and Tigris continued into the early Republican period, as the rivers continued to be handled separately. When the discussions surrounding the river basin mounted due to the construction of the Keban Dam, interest remained limited to the Euphrates basin and how to handle its lower parts after the construction of the dam. However, even though state actors prioritized harnessing the Euphrates River, they also subjected the Tigris to reconnaissance surveys. In 1968, two years after the preparation of the “Reconnaissance Report for the Lower Euphrates Basin,” the FPGA prepared a “Reconnaissance Report for the Tigris Basin” (*Dicle Havzası İstikşaf Raporu*). In the 1970s, these reports on the Lower Euphrates and the Tigris merged into GAP after Turkish expert-bureaucrats claimed that the rivers shared a common basin, to be called the Euphrates-Tigris basin.

The most immediate implication of this claim of a single basin was that the new project region overlapped with the entire region of Southeast Anatolia, the region of territorial disputes and armed conflict. Consequently, state investment in this Kurdish-dominated region was a thorny issue and needed legitimation in the eyes of Turkish nationalists. Official discourse tried to manage this problem by drawing a distinction between the Kurdish population in Southeast Anatolia and the PKK, by denying the existence of local support for the latter. The promise of the state was clear: to sow the seeds of peace through the implementation of GAP (see, e.g., Yılmaz 1987; Balbay 2009). Nevertheless, the project would ultimately contribute to the formation of new spaces that bolstered the military power of the PKK. The way the basin was defined as a single basin created tension and even the threat of war among the states adjacent to the Euphrates and the Tigris, namely Iraq, Syria, and Turkey—relations that were already tense due to the construction of the Keban Dam. In the first bilateral meetings with Iraq in 1964, Turkey promised Iraq that the minimum flow downstream from Keban would be 350 m^3^/s. In this meeting, Turkey also suggested that the rivers belong to a common watercourse, which means Iraq could compensate for the decrease in the flow of the Euphrates by transferring water from the Tigris (Williams 2012).

As defined by the UN Watercourse Convention, watercourses are a system of ground and surface waters. This is a narrower definition than that of river basin, which refers also to land territories (Rieu-Clarke et al. 2012: 77–8). In this regard, the articulation of the watercourse by Turkish authorities can be seen as the step before making the single basin claim. When experts, mostly technocrats from Turkey, made the single basin claim in the 1970s, they utilized the natural qualities of the basin to make their point. Although the Euphrates and the Tigris have separate drainage basins within the borders of Turkey, they asserted that the rivers meet in the Shatt-al-Arab before emptying into the Persian Gulf; there is no natural barrier between the two rivers; and it is very difficult to demarcate the watershed boundaries in Iraq near the confluence point (Bilen 2000; Kibaroğlu & Ünver 2000). Yet, while relevant actors in Turkey used the proximity of the rivers in Iraqi territory, Iraq and Syria vociferously rejected the claim, since the rivers flow separately for most of their path. Syria objected to the single basin claim on the grounds that there was no surplus in the Tigris to be transferred to the Euphrates and the rivers merged downstream of Syria. Also, while Turkish authorities gave the example of the Iraqi Tharthar Canal, which connects two rivers and allows water transfers, to support their single basin claim, Iraqi authorities also rejected this reasoning because the canal was manmade and functioned as a flood channel. These different views on rivers turned into long-lasting disputes between the riparian states. International law was of little use to solving these disputes, because all parties could find a principle supporting their claims, which turned the issue into a matter of political will (Lorenz & Erickson 1999).

Despite strongly supporting the single basin claim, Turkish experts and politicians did not specify the meaning and reasons behind such a claim in terms of river management. Yet, the conflicts between the scientific claims about the Euphrates and the Tigris were congruent with the differences in the interests of the groups making those claims. According to the Association of International Law, the respective contribution of riparian states to a given river was the major factor in determining the share in the use of the water resources (Bilen 2000: 36). When the rivers were considered separately, Turkey appeared to be the major contributor to the Euphrates, which means the headwaters or tributaries of the Euphrates are mostly within the borders of the Turkish state. Nevertheless, the same does not hold for the Tigris. As Table 1 demonstrates,^4^ Turkey contributes to the flow of the Tigris less than Iraq does, which made the latter uncompromising in any agreement about the use of the Tigris. Yet, by claiming that the Euphrates and the Tigris form a joint basin, Turkey compensated for its relatively limited contribution to the latter and gained an upper hand in harnessing it as well (Harris & Alatout 2010).

**Table 1:** Average percentage of annual flows and contributions of riparian states. *Source:* Bilen (2000)

River	Average Annual Flow (in BCM)	Water Contribution by Country (in BCM (km^3^))		
		Turkey	Syria	Iraq
Euphrates	35	31.6 (90%)	3.4 (10%)	0
Tigris	52.7	21.3 (40%)	0	31.4 (60%)

This compensation, however, cost Turkey a great deal, in both political and economic terms. In terms of politics, the relations between riparian states grew worse over the course of GAP and shaped the war within Turkey’s borders. In its 1992 report, the London-based International Institute for Strategic Studies warned Turkey about the political retaliation that could come from Syria and Iraq because of Turkey’s use of the Euphrates and the Tigris. The report stated that in the late 1980s, PKK camps were already being transferred to Syria and PKK militants infiltrated the Turkish border from those camps (*Cumhuriyet* 1992). Several factors put ethnic warfare in Southeast Turkey on the agenda of the riparian states, such as the 1980 military coup, the military regime’s violence against Kurds, the influx of Kurdish refugees into Turkey due to the Iran-Iraq War (1980–1988), and the establishment of a semi-autonomous Kurdish government in northern Iraq. While the PKK emerged not only as a response to state violence but also to the conditions in Kurdish society, state violence in the post-coup period deepened insecurity and anger among the Kurdish population and led to higher levels of recruitment into the PKK (Tezcür 2015). In 1984, one year after Turkey initiated GAP’s largest dam project on the Euphrates, the Syrian-controlled Bekaa Valley was already serving as an initial launching pad for the PKK’s incursion into Southeastern Turkey. At a moment of intensifying warfare, Turkey’s prime minister visited the Syrian president, and they reached an agreement in 1987 for a minimum flow of water in exchange for Syria’s decreased support for the PKK. However, a few years later, the filling of the reservoir of the GAP’s largest dam on the Euphrates, the Atatürk Dam, revived these tensions that even led to the sheltering of PKK leader Abdullah Öcalan in Syria. This Syrian support for the PKK lasted until the late 1990s, when Turkish troops directly threatened Syria with war (Langer 2009). So, the worsening relations between the riparian states served the interests of the Kurdish armed struggle, which in turn, made Turkey double its military expenditure between the late 1980s and early 1990s.

While the increase in Turkey’s military expenses meant less investment for GAP, the finances of GAP were also influenced directly by the tensions between Turkey and the other riparian states. Not being able to solve its water problems with Turkey, Syria and Iraq put pressure on international financial organizations and contractors. They wrote furious letters to foreign investors and constructors to make them stop their involvement in the project (Warner 2008: 283). This resulted in the withdrawal of many organizations, including the World Bank, from supporting the projects, which meant that Turkey was forced to finance the projects domestically. This caused significant delays in the construction of the infrastructure projects, especially those related to irrigation development. Thus, just as existing social/political relations affected decisions about the scale and the administration of the project, those decisions, in turn, shaped the relations which played important roles in translating the plans into concrete constructions.

All in all, Turkey’s claim to a conjoined basin had important effects on its relations with the wider world. This shows how something deemed completely natural, the river basin, was actually an entanglement of political and economic interests, negotiations, and contestations. With the single basin claim, state elites in Turkey turned a politically contentious region into a region of development, an act that could be interpreted in two conflicting ways: first, as an act of colonization against the Kurdish territories for the purpose of national economic development; second, as an act of peacebuilding through the economic development of the Kurdish territories. For example, on the one hand, the leader of the PKK, Abdullah Öcalan, described Southeastern Turkey as a colony, and claimed that GAP is for transferring the resources of Kurdish lands to other parts of the country (Özcan 1999). On the other hand, state authorities claimed that GAP would sow the seeds of peace by turning the land-mined areas into agricultural fields. However, despite the prominence of the Kurdish conflict in the creation of GAP, the official discourse avoided any political confrontation with the Kurdish population (Bilgen 2017). Even the project documents, which included a great deal of information about the regional population, made no reference to the Kurdish population, their nation, and their culture. Rather, references to them were invariably about the ancient populations of the region that had established civilizations in the region between the Euphrates and the Tigris, in what was known as Mesopotamia. There, the promise of GAP was clear: returning to Mesopotamia its previous prosperity.

## Conclusion

In Ancient Greek, Mesopotamia means between the rivers. Historically, the word signifies the region between the Euphrates and the Tigris, whose territory extends mostly within present-day Iraq but also to Southeastern Turkey. Given that Mesopotamia was home to early civilizations such as the Sumerians and the Acadians, GAP’s promise was to return the region to its fertile past as the “cradle of civilization.” This promise was supported by the single basin claim and invited the population to imagine a prosperous future by preventing discussions about the existence of a Kurdish population in the region. The technical claims of a single basin and references to Mesopotamia thus contributed to making the scale of the river basin and legitimizing the act of turning a politically contentious region into a region of infrastructural transformation. Nevertheless, the soils of Mesopotamia were far from fertile when GAP was planned. While the trademark of Mesopotamia was its rivers, namely its water potential, the region also had a water problem, due to irregular and low rainfall. Thus, the past(s) and future(s) of Mesopotamia were marked by two conflicting realities: the abundance and scarcity of water. This conflict could be solved by carrying water from where it was abundant to where it was lacking, which meant using river water to irrigate the lands of Mesopotamia. The importance of integrating land and water, one of the main ideas behind IRBP, had already been acknowledged in Mesopotamia in ancient times (Dağ & Göktürk 1994: 9). As the narratives around GAP suggested, the Assyrian King Nimrod was the first person foreseeing today’s GAP: he forced 10,000 slaves to divert the flow of the Euphrates toward Harran, one of the ancient cities of Mesopotamia (Saraç 1994). Centuries later, Demirel was framed as the great leader who realized Nimrod’s dreams (ibid.). His motto included an implicit reference to these past dreams: “It is not mountains but the epochs we are digging” (cited in *Cumhuriyet* 1994).

This kind of discourse clearly spoke to ideas of national strength and power, and was appealing to the Turkish nationalists for whom large investments in Kurdish-populated regions remained questionable. In the midst of economic and political constraints, the project’s legitimacy was vulnerable—in the circles of politicians and the Turkish population alike, requiring particular discourses through which the changing economic conditions and the suspicion of separatism were challenged. This work fell upon both politicians and experts sharing or feigning Demirel’s enthusiasm: while state experts continued producing technical studies to come up with a more feasible plan, high-ranking bureaucrats framed the plan as a national project that would be the product of national actors to benefit the national population rather than merely the population of Eastern Turkey.

The process of technology transfer including the transfer of knowledge and administrative frameworks depends not only on technical values and parameters but also on cultural values and political conditions. Technical language cannot easily be separated from the material context within which it was created. This means that knowledge is modified and enriched as it travels between different contexts with different geographical characteristics, organizational structures, and social relations. When the distinction between TVA and IRBP (so-called TVA idea), and the simultaneous multiplicity of IRBP projects are acknowledged, it becomes easier to see the politics behind the dissemination of IRBP. This kind of approach pushes the researcher to focus on the construction of expertise networks and the politics involved in them. In this regard, the emphasis on the distinction between TVA and IRBP stands as a starting point for demonstrating how techno-scientific arguments are utilized to conceal the cultural and political dimensions of river basin development. The power of this line of argumentation becomes even more apparent in the context of the politics of the nation-state, or more particularly within ethnic conflicts—as was the case in the Turkish context.

Since the early years of the Republic, Southeast Turkey has been a problem space for Republican leaders, due to the region’s large Kurdish population, which did not conform to the rules and regulations of the Turkish nation-state. Every act delineating Turkey into regions, even for educational purposes, received pushback on the grounds that it could have political implications or threaten the unity of the nation-state by supporting separatist motivations. The situation was different, albeit relatively, for the DSİ’s division of the country into river basins, because this division could more easily lend itself to scientific explanations and developmental promises. The one basin claim played a similar role by suddenly translating a political region into a technical one. Moreover, the claim was well-aligned with the discourses on Mesopotamia, further supporting the promises of development and veiling the more recent political past of the region. In the end, the story of the Euphrates and the Tigris basins—ending in the Euphrates-Tigris basin—reveals that techno-scientific practices are about not only knowledge and skills, but also the ways in which they are organized, negotiated, and modified in a way that resonates with the histories, politics, and material conditions of a given region and its relationship with other relevant scales. In this light, technological development can be seen as a process through which the political, social, and technical are made legible to each other.

## Footnotes

1 A few early examples include the Damodar Valley Corporation in India (Goodall 1945), the Snowy Mountains Hydroelectric Authority in Australia (Byrne 2000), the Yangtze Valley Authority in China (before the establishment of the People’s Republic of China), the Helmand Valley Authority in Afghanistan (Cullather 2002), the Corporacion Venezolana de Guayana in Venezuela (Martin 1970), the Khuzestan Water and Power Authority in Iran, and the Autonomous Regional Development Corporation of the Cauca Valley in Columbia (Escobar 1995).

2 For more detailed information about the colonial roots of river basin planning, see Lagendijk (2019).

3 Since Keban was considered a hydropower project, EİE remained the body responsible even after the establishment of DSİ.

4 Just as with adding the rivers’ annual flows together, the act of measuring relates to the ways of seeing nature through particular lenses. This table stands not as an acknowledgment of this act of measuring, but as an invitation to see what is behind the measurement when one wears those lenses. Otherwise, the numbers presenting the annual flows change from one source to another, mostly due to the fluctuations in the flow of the rivers.

## References

[CR1] Adalet B (2018). Hotels and highways: the construction of modernization theory in cold war Turkey.

[CR2] APA (Alaska Power Authority) (1982). Technical proposal binder 2.

[CR3] Ardıçoğlu N (1963). Keban Barajı ve Aşağı Fırat Havzası Kalkınması Hakkında Muhtıra.

[CR5] Balbay, Mustafa 2009. Mayınlı araziye barış tohumu. *Cumhuriyet *(9 June 2009*)*.

[CR6] Bijker WE, Goodin RE, Tilly C (2006). Why and How Technology Matters. The Oxford Handbook of Contextual Political Analysis.

[CR7] Bilen Ö (2000). Turkey and water issues in the middle east.

[CR8] Bilgen, Arda 2017. *Demystifying the (Post‑) Politics of Southeastern Anatolia Project (GAP)*. PhD Thesis, Universität Bonn.

[CR10] Bilgen A (2019). The southeastern Anatolia project (GAP) in Turkey: an alternative perspective on the major rationales of GAP. Journal of Balkan and Near Eastern Studies.

[CR11] Bochenski F, Diamond W (1950). TVAs in the middle east. The Middle East Journal.

[CR12] Bozarslan H (2009). Kurds and the Turkish State. The Cambridge History of Turkey.

[CR13] Bozdoğan S, Isenstadt S, Rizvi K (2008). Democracy, development, and the americanization of Turkish architectural culture in the 1950s. Modernism and the middle east: architecture and politics in the twentieth century.

[CR14] Byrne G (2000). Schemes of nation: a planning history of the snowy mountains scheme.

[CR22] Çelik F (2019). Entegre nehir havzasını yönetme deneyimlerinin karşılaştırmalı analizi: ABD ve Türkiye örnekleri. Mehmet Akif Ersoy Üniversitesi Sosyal Bilimler Enstitüsü Dergisi.

[CR15] Cullather N (2002). Damming Afghanistan: modernization in a buffer state. The Journal of American History.

[CR16] *Cumhuriyet* 1949. Ortadoğu’nun Kalkınması. *Cumhuriyet *(25 August 1949).

[CR17] *Cumhuriyet* 1955a. Siyasal Bilgiler Fakültesinde açılan sergi. *Cumhuriyet *(29 January 1955).

[CR18] *Cumhuriyet* 1955b. Westinghouse müessesesile imza edilen mukaveleler. *Cumhuriyet* (24 June 1955)*.*

[CR19] *Cumhuriyet* 1962. Hükümet ve planlama dairesinin muhalefetine rağmen tahsisat çıkarıldı. *Cumhuriyet* (21 Janurary 1962)*.*

[CR20] *Cumhuriyet* 1992. PKK Su ile Bağlantılı. *Cumhuriyet *(21 May 1992).

[CR21] *Cumhuriyet* 1994. Fırat Harran’a kavuştu. *Cumhuriyet* (10 November 1994).

[CR23] Dağ R, Göktürk A (1994). GAP yeniden yapılanmalıdır.

[CR24] Embid A (2003). The transfer from the ebro basin to the mediterranean basins as a decision of the 2001 national hydrological plan: the main problems posed. International Journal of Water Resources Development.

[CR25] Engerman DC, Unger CR (2009). Introduction: towards a global history of modernization. Diplomatic History.

[CR26] Eriç, Daniyal 1962. Keban Barajı. *Cumhuriyet *(4 February 1962).

[CR27] Erken A (2019). America and the making of modern Turkey: science, culture and political alliances.

[CR28] Escobar A (1995). Encountering development: the making and unmaking of the third world.

[CR29] Geray C (1965). Toplum Kalkınmasında Halk Katılışları, Destekli İmece ve emanet Usulü Uygulama. Ankara Üniversitesi SBF Dergisi.

[CR30] Geray C (1997). Bölgesel gelişme için planlama ve örgütlenme. Ankara Üniversitesi SBF Dergisi.

[CR31] Gilman N (2003). Mandarins of the future: modernization theory in cold war America.

[CR32] Goodall MR (1945). River valley planning in India: the damodar. The Journal of Land & Public Utility Economics.

[CR33] Gorgas JT (2009). “The Shared Political Production of ‘the East’ as a ‘Resistant’ Territory and Cultural Sphere in the Kemalist Era, 1923–1938.”. European Journal of Turkish Studies. Social Sciences on Contemporary Turkey.

[CR34] Harris LM, Alatout S (2010). Negotiating hydro-scales, forging states: comparison of the upper Tigris/Euphrates and Jordan river basins. Political Geography.

[CR35] Hartmann H (2020). Eigensinnige Musterschüler: Ländliche Entwicklung und internationales Expertenwissen in der Türkei (1947–1980</i>).

[CR36] Hirst SJ, İşçi O (2020). Smokestacks and pipelines: Russian-Turkish relations and the persistence of economic development. Diplomatic History.

[CR37] Hoag HJ (2006). Transplanting the TVA? International contributions to postwar river development in Tanzania. Comparative Technology Transfer and Society.

[CR38] Husain FH (2021). Rivers of the sultan: the tigris and euphrates in the ottoman empire.

[CR40] İşçi O, Boyar E, Fleet K (2022). Interwar Territoriality and soviet-Turkish convergence across the Aras river. Borders, boundaries and belonging in post-ottoman space in the Interwar period.

[CR39] Ives RH, Bochar RM (2002). From the colorado river to the nile and beyond: a century of reclamation’s international activities.

[CR41] Kangarani H (2005). Forestry outlook study for west and central Asia: euphrates and tigris watershed. Economic, social and institutional aspects of forest in an integrated watershed management.

[CR42] Kasaba R (2000). Hard times in Turkey. New Perspectives on Turkey.

[CR43] Keskin-Kozat B (2011). Reinterpreting Turkey’s marshall plan: of machines, experts, and technical knowledge. American Turkish encounters: politics and culture, 1830–1989.

[CR44] Keskin-Kozat B, Cangül Örnek, Üngör Ç (2013). Negotiating an institutional framework for Turkey’s Marshall Plan: the conditions and limits of power inequalities. Turkey in the Cold War.

[CR45] Kibaroğlu A, Ünver IO (2000). An institutional framework for facilitating cooperation in the euphrates-tigris river basin. International Negotiation.

[CR46] Klingensmith D (2007). One valley and a thousand: dams, nationalism, and development.

[CR47] Köymen, Nusret 1948. Amerika sel felaketini elektrik nimetine çevirmede bize örnek olmalıdır. *Cumhuriyet* (26 February 1948).

[CR48] Kranakis E (2021). Writing technology into history. Technology and Culture.

[CR49] Krige J, Krige J (2019). Introduction. How knowledge moves: writing the transnational history of Sscience and technology.

[CR50] Kutan R (2001). Açılış Konuşması [Opening Speech]. Her Yönüyle GAP-Sempozyum.

[CR51] Lagendijk V (2018). From American south to global south: the TVA’s experts and expertise, 1933–1998. Work in progress: economy and environment in the hands of experts.

[CR52] Lagendijk V (2019). Streams of knowledge: river development knowledge and the TVA on the river mekong. History and Technology.

[CR53] Langer A (2009). Hydro Wars: The Struggle for Water and Survival in the Euphrates-Tigris River Basin. Journal of Politics and Society.

[CR54] Lorenz FM, Erickson EJ (1999). The euphrates triangle: security implications of the southeastern Anatolia project.

[CR55] Luke C (2019). A pearl in peril: heritage and diplomacy in Turkey.

[CR56] Martin, Roscoe C. 1970. *TVA and International Technical Assistance: A Report to the Board of Directors and the General Manager Tennessee Valley Authority*. Syracuse: Consultant Report.

[CR57] Molle F (2009). River-basin planning and management: the social life of a concept. Geoforum.

[CR58] Mukhtarov, Farhad 2009. *The Hegemony of Integrated Water Resources Management: A Study of Policy Translation in England, Turkey, and Kazakhstan*. PhD Thesis, Department of Environmental Sciences and Policy, Central European University.

[CR59] Nadi, Yunus 1938. Hakikatler var ki hayallerden çıkar! Bizim, Ankara’yı denize bağlıyan kanallarımız ulaşılmaz bir hayal değildir. *Cumhuriyet *(16 July 1938).

[CR60] Özcan N (1999). PKK (Kürdistan İşçi Partisi) tarihi, ideolojisi ve yöntemi.

[CR61] Rieu-Clarke, Alistair, Ruby Moynihan, and Bjørn-Oliver Magsig 2012. *UN Watercourses Convention: A User’s Guide*. IHP-HELP Centre for Water Law, Policy and Science (under the auspices of UNESCO). ISBN: 9780957260306.

[CR62] Rustow D (1987). Turkey, America’s forgotten ally.

[CR63] Saraç, Mehmet 1994. Harran insanı inandı, kazandı. *Cumhuriyet* (14 November 1994).

[CR64] Smith S-N (2022). Metrics of modernity: art and development in postwar Turkey.

[CR65] Sneddon C (2015). Concrete revolution.

[CR66] Stahl, Dale 2014. *The Two Rivers: Water, Development and Politics in the Tigris-Euphrates Basin, 1920–1975*. Phd Thesis, Columbia University.

[CR67] Stahl D, İnal O, Turhan E (2019). A technopolitical frontier: the Keban dam project and southeastern Anatolia. Transforming socio-natures in Turkey: landscapes, state and environmental movements.

[CR68] Taşkın O (2015). Türkiye’nin ilk dev barajı Keban 41 Yaşında.

[CR69] Teclaff L (1967). The river basin in history and law.

[CR70] Tekeli İ (2008). Türkiye’de bölgesel eşitsizlik ve bölge planlama yazıları.

[CR71] Tezcür GM (2015). Violence and nationalist mobilization: the onset of the Kurdish insurgency in Turkey. Nationalities Papers.

[CR72] Tunç TE, Tunç G (2022). “‘A Light Bulb in Every House’: The Istanbul General Electric Factory and American Technology Transfer to Turkey.”. Technology and Culture.

[CR73] Turgut H (1992). Bir liderin yükselişi (1962–1971).

[CR74] Turgut H (2000). GAP ve Demirel.

[CR75] Turgut H (2005). Bir ömür suyun peşinde.

[CR77] Türkiye Büyük Millet Meclisi (1962). Keban Barajı ve Aşağı Fırat Havzası Kalkınma Projesi Hakkında Millet Meclisi Adına Araştırma Komisyonu.

[CR76] Türkiye Büyük Millet Meclisi 1958 (February 26). *Tutanak Dergisi (Term 11, Year 1, Sitting 47).* Ankara: TBMM.

[CR78] Türkiye Büyük Millet Meclisi 1964 (July 1). *Tutanak Dergisi (Term 17, Year 3, Sitting 49).* Ankara: TBMM.

[CR82] Vali AF (2019). Bridge across the Bosporus: the foreign policy of Turkey.

[CR79] Warner J (2008). Contested hydrohegemony: hydraulic control and security in Turkey. Water Alternatives.

[CR83] Wescoat J, Fishman R (2000). Watersheds in regional planning. The American planning tradition: culture and policy.

[CR80] White GF (1957). A Perspective of River Basin Development. Law & Contemporary Problems.

[CR81] Williams P, Vajpeyi DK (2012). Euphrates and Tigris waters: Turkish-Syrian and Iraqi relations. Water resource conflicts and international security: a global perspective.

[CR84] Yeğen M (2011). Son Kürt İsyanı.

[CR85] Yıldız, Dursun 2014. *60. Yılında Su Filomuzun Amiral Gemisi: DSİ*. Retrieved in March 29, 2021 from https://hidropolitikakademi.org/uploads/wp/2014/09/DS%C4%B0-Tarihi.pdf.

[CR86] Yılmaz, Turan 1987. Mayınlı alanlar tarla olacak. *Cumhuriyet* (14 March 1987).

